# Evaluation of Drug Use Pattern in Emergency Department of Dilchora Referral Hospital, Dire Dawa, Ethiopia

**DOI:** 10.1155/2020/4173586

**Published:** 2020-02-25

**Authors:** Kirubel M. Mishore, Nigatu A. Bekele, Tsegaab Y. Yirba, Tsion F. Abone

**Affiliations:** ^1^Department of Clinical Pharmacy, School of Pharmacy, College of Health and Medical Sciences, Haramaya University, Harar, Ethiopia; ^2^Department of Pharmacy, College of Health and Medical Sciences, Dilla University, Dilla, Ethiopia; ^3^Independent Researcher, Wolkite, Ethiopia

## Abstract

**Background:**

Drug use evaluation is a system of continuous, systematic, criteria-based drug evaluation that ensures the appropriate use of drugs. Rationalization of drug therapy in emergency medicine would be useful in managing the broad array of conditions that present for emergency care. High-quality drug utilization is associated with the use of a relatively limited number of essential medicines. The World Health Organization developed core drug use indicators for conducting drug utilization studies in healthcare setting. WHO core drug use indicators including prescribing indicators, patient care indicators, and health facility indicators are used nowadays.

**Objective:**

The aim of this study was to evaluate the drug use pattern in the Emergency Department of Dilchora Referral Hospital, Dire Dawa, Ethiopia, 2018.

**Methods:**

A retrospective cross-sectional descriptive study was conducted in the emergency department (ED) of Dilchora Referral Hospital from July 20 to August 19, 2018, using structured data collection format.

**Result:**

Out of 344 prescriptions analyzed, a total of 753 medications were prescribed. The average number of drugs per prescription was 2.19. Of drugs prescribed, 685 (90.97%) were in their generic names. Antibiotics were prescribed in 95 (27.62%) of encounters, and injections were prescribed in 154 (44.77%) of encounters. Among 753 medications prescribed, the name and strength of drugs are indicated in 100% and 95.22%, respectively. 679 (90.17%) of drugs were prescribed from the essential drug list of Ethiopia.

**Conclusion:**

The findings of this study revealed that the drug utilization pattern was not optimal in accordance with the standard values of WHO prescribing indicators. Some of the prescribing indicators like overprescribing of antibiotics and injections were a problem. Therefore, it is very imperative for the concerned stakeholders and healthcare providers to work toward ensuring drug use according to the standard.

## 1. Introduction

Drug use evaluation is a system of continuous, systematic, criteria-based drug evaluation that ensures the appropriate use of drugs. It is a method of obtaining information to identify problems related to drug use, and if properly developed, it also provides a means of correcting the problem and thereby contributes to rational drug therapy. It improves the quality and cost effectiveness of drug use and thereby improves patient care. It is a mandated multidisciplinary quality management program that focuses on evaluating medication effectiveness and improving patient safety [1, 2].

Rationalization of drug therapy in emergency medicine would be useful in managing the broad array of conditions that present for emergency care [3]. Physicians often face challenges in selecting, initiating, and individualizing appropriate drug therapy for patients in the emergency room [4].

Worldwide, more than 50% of all medicines are prescribed, dispensed, or sold inappropriately, while 50% of patients fail to take them correctly. Moreover, about one-third of the world's population lacks access to essential medicines. Polypharmacy and failure to prescribe in accordance with clinical guidelines are some of the irrational use of drugs [5].

High-quality drug utilization is associated with the use of a relatively limited number of pharmaceutical products (essential medicines). Essential medicines are described as “those that satisfy the priority health care needs of the population. They are intended to be available within the context of functioning health systems at all times in adequate amount and appropriate dosage forms with assured quality” [6–8].

Despite the potential health impact of essential drugs and despite substantial spending on drugs, lack of access to essential drugs, irrational use of drugs, and poor drug quality remain serious global public health problems [9].

WHO developed core drug use indicators for conducting drug utilization studies in healthcare setting. The core drug use indicators are more informative, more feasible, less likely to fluctuate over time and place. The core drug use indicators were developed to measure performance in three general areas related to the rational drug use (RDU). WHO core drug use indicators include prescribing indicators, patient care indicators, and health facility indicators [10–12].

Following the formation of International Network for the Rational Use of Drugs (INRUD) to conduct multidisciplinary intervention research to promote the rational use of medicines in 1989, WHO and INRUD designed standard methodology for selected drug use indicators in health facilities. A small number of core indicators are recommended, and they are highly standardized and grouped as prescribing indicators (average number of drugs per encounter, percentage of drugs prescribed by generic name, percentage of encounters with an antibiotic prescribed, percentage of encounters with an injection prescribed, and percentage of drugs prescribed from the essential drug list (EDL)), patient care indicators (average consultation time, average dispensing time, percentage of drugs actually dispensed, percentage of drugs actually labeled, and patient knowledge of how to take the drug), and facility indicators (availability of copy of the health facility's medicines list and availability of key essential medicines). By increasing access to essential drugs and their rational use, we could improve health status and secure development gains [10, 13, 14].

The principal goal of drug utilization study is to facilitate the rational use of drugs in populations. RDU generally covers appropriate prescribing, appropriate dispensing, and appropriate patient use of medicines for the diagnosis, prevention, mitigation, and treatment of diseases. To enhance RDU, the patient should receive medicines appropriate to their healthcare conditions, at optimum doses and sufficient time, as well as at the cost that the individual and the community can afford [11].

Promoting rational drug use is multifactorial and needs collaboration. Though different studies were conducted at different corners of the country on drug use assessment, less is known about drug use in emergency wards. Consequences of facility-related factors like availing vital drugs for emergency cases might be too different from other drugs used in cases treated at outpatient department. Therefore, the aim of this study was to evaluate the drug use pattern in the Emergency Department of Dilchora Referral Hospital, Dire Dawa, Ethiopia, 2018.

## 2. Materials and Methods

### 2.1. Study Area and Period

The study was conducted at ED of DRH, Dire Dawa Administration health bureau, Dire Dawa, Eastern Ethiopia, from July 20 to August 19, 2018. Dire Dawa is one of the two chartered cities in Ethiopia and is located 515 km far from Addis Ababa in the east and 313 kilometers to the west of Port Djibouti. Dilchora Referral Hospital is found in Dire Dawa Administration, in the North East of the city. It is one of the government referral hospitals in the eastern region of country, established in 1952 E. C. The ED of the hospital represents an important platform for conducting the drug utilization pattern as patients present with a wide range of diseases in acute form and the drug use is quite extensive.

### 2.2. Study Design

A retrospective cross-sectional descriptive study was conducted to evaluate the drug use pattern in ED of DRH using WHO drug use indicators.

### 2.3. Population

All dispensed prescriptions at the emergency department of DRH were source population, whereas those randomly selected prescriptions from prescribed drugs in the emergency department of DRH during the study period were study population.

### 2.4. Inclusion and Exclusion Criteria

All legible prescriptions in ED irrespective of patients' age, sex, and diagnosis were included, and prescriptions with no medicine (equipment, supplies, and reagents) were excluded from the study.

### 2.5. Sampling Size Determination and Sampling Techniques

Sample size was determined by using a single proportion formula for cross-sectional study and taking the proportion of the prevalence of rate of drug utilization 50% with a confidence level of 95% and degree of precision of 5%. By using the formula, the sample size was found to be 384. Since our source population was less than 10,000, finite population correction was used. Then adding 5% nonresponses, the final sample size was 344. Three-hundred forty four prescriptions were randomly selected from drugs prescribed in the emergency department of DRH.

### 2.6. Data Collection Tools and Procedure

Data were collected using structured format through reviewing prescription papers by two trained pharmacists. The format was pretested and subsequent correction was done, and the data collectors were trained on the data collection technique. The format included sociodemographic variables, diagnosis, and drug information. The collected data were reviewed and checked for completeness before data analysis.

### 2.7. Data Processing and Analysis

The collected data were coded, entered, cleaned, and analyzed by using SPSS version 21. Descriptive analysis such as percentage and frequency distribution was made. WHO prescribing indicators were calculated, including the average number of drugs per encounter, percentage of drugs prescribed by generic name, percentage of drugs prescribed from the essential drug list, and percentage of encounters during which an antibiotic was prescribed. WHO facility indicators and some patient care indicators were also computed. Finally, the result was interpreted and presented by tables and graphs.

### 2.8. Ethical Consideration

Initially ethical clearance was obtained from the School of Pharmacy, College of Health and Medical Sciences, Haramaya University. Patients' data were accessed upon the approval of medical director of DRH. Confidentiality was ensured during the data collection; the information gathered from this study was not disclosed to others.

### 2.9. Operational Definitions

#### 2.9.1. Average Number of Drugs per Encounter

The average number of drugs prescribed per encounter is calculated to measure the degree of polypharmacy. It was calculated by dividing the total number of different drug products prescribed by the number of encounters surveyed. Combinations of drugs prescribed for one health problem will be counted as one.

#### 2.9.2. Percentage of Drugs Prescribed by Generic Name

Percentages of drugs prescribed by generic name are calculated to measure the tendency of prescribing by generic name. It was calculated by dividing the number of drugs prescribed by generic name by the total number of drugs prescribed, multiplied by 100.

#### 2.9.3. Percentage Encounter with Injection Prescribed

Percentage of encounters with an injection prescribed is calculated to measure the overall level use of commonly overused and costly forms of drug therapy. It was calculated by dividing the number of patient encounters in which an injection was prescribed by the total number of encounters surveyed, multiplied by 100.

#### 2.9.4. Percentage of Antibiotic per Encounter

Percentage of encounters in which an antibiotic prescribed is calculated to measure the overall use of commonly overused and costly forms of drug therapy. It was calculated by dividing the number of patient encounters in which an antibiotic was prescribed by the total number of encounters surveyed, multiplied by 100.

#### 2.9.5. Percentage of Drugs Prescribed from Essential List or Formulary

Percentage of drugs prescribed from an EDL is calculated to measure the degree to which practices conform to a national drug policy as indicated in the national drug list of Ethiopia. Percentage was calculated by dividing the number of products prescribed which are in essential drug list by the total number of drugs prescribed, multiplied by 100.

## 3. Results

### 3.1. Sociodemographic Characteristics of the Patients

A total of 344 prescriptions were analyzed in the study. Out of 344 prescriptions, majority of them (211; 61.34%) were male. The mean age of participants was 26 ± 16.1 (Table 1).

Sixteen (4.65%) of the study participants had atleast one comorbid case. Eight (2.33%) patients had hypertension as a comorbid while five (1.45%) were diabetic patients.

**Figure 1:**
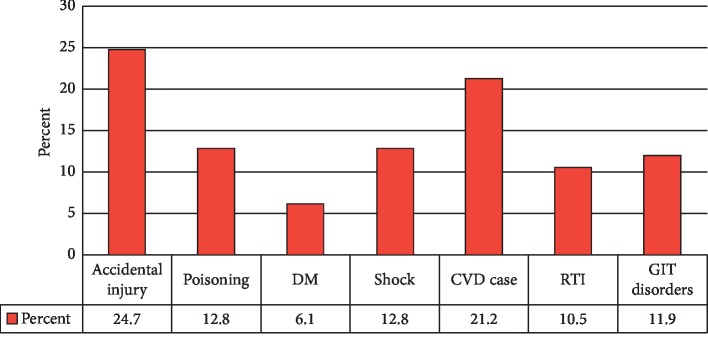
Pattern of distribution of diagnosis among patients admitted at the emergency department of Dilchora Referral Hospital, July 20-August 19, 2018.

The main reason of admission to the Emergency Department was accidental injury mainly vehicle accident (24.7%) followed by cardiovascular complication including stroke (21.2%) (Figure 1).

Most of these accidental injuries were as a result of bicylic and tricyclic motor vehicles, which were mostly handled by young individuals justifying the young mean age of patients participated in the study. Poisoning was a third leading cause of hospital admission (12.8%).

### 3.2. General Prescribing Pattern

A total of 753 individual drugs were prescribed for 344 drug encounters, giving an average of 2.19. Among 753 drugs prescribed, 245 (32.54%), 208 (27.62%), and 95 (12.62%) were cardiovascular, antibiotics, and NSAIDS drug classes, respectively (Table 2).

Regarding information related to prescribed drugs, drugs' names were indicated in all of them. 717 (95.22%), 700 (92.96%), and 685 (90.97%) of drugs had strength, frequency, and dose indicated as shown in Table 3.

### 3.3. Prescribers' Indicators

Among 753 drugs prescribed, 685 (90.97%) were with generic nomenclature. Of all 344 encounters, 95 (27.62%) and 154 (44.77%) encounters were appeared with antibiotics and injection, respectively (Table 4).

253 (73.55%) of the prescriptions contained two or more drugs. The maximum number of drugs per encounter is five (Table 5).

### 3.4. Facility Indicators

The study found that the hospital has a copy of the EDL. 679 (90.17%) of total drugs prescribed were from the EDL. Only 174 (25.63%) of drugs prescribed from the EDL was available at the hospital during the study period. Among 25 drugs selected as key essential drugs, 16 (64%) were available.

### 3.5. Patient Care Indicators

Prescription registration book assessment showed that, among 753 drugs prescribed, 246 (32.67%) were actually dispensed from the institution. Other patient care indicators (average consultation time and average dispensing time) were not measured as the study was purely retrospective in nature.

## 4. Discussion

The average number of drugs per prescription was 2.19. This finding was higher than the standard derived as ideal (1.6–1.8) [7, 11]. The finding was also higher than the study done at Hawassa University Hospital, Jimma Hospital, and Dilla University Referral Hospital where the average number of drugs prescribed per encounter was 1.9, 1.76, 1.813, and 1.76, respectively [15–18]. In this study, the average number of drugs per encounter showed the presence of overprescribing in hospitals, polypharmacy. A high average number of drugs might be due to lack of therapeutic training of prescribers, or shortage of therapeutically correct drugs.

The finding of this study was lower than studies at Oman, Pakistan, Saudi Arabia, and Ayder Referral Hospital of Northern Ethiopia, which showed that the average number of drugs per prescription was 3.2, 2.8, 2.4, and 2.61, respectively [19–22]. The low values might be due to there is constraint in the availability of drugs, or prescribers have appropriate training in therapeutics.

The percentage of drugs prescribed by generic name in this study was 91%. This finding was lower than the standard derived to serve as ideal (100%) [7, 11] and the studies conducted at Hawassa University Hospital (98.7%) and Dilla University Referral Hospital (85.33%) [15, 16]. It is higher than the studies carried out at Pakistan, Saudi Arabia, and Jimma Hospital, where the percentage of drugs prescribed by generic names was 56.6%, 61.2%, and 87.1%, respectively [17, 20, 21].

The percentage of encounters in which antibiotics were prescribed in this study was 27.62%, which was high compared to the standard (20.0%–26.8%) derived to be ideal [7, 11] but lower than the study at Ayder Referral Hospital of Northern Ethiopia (32%), national baseline study (58.10%), and Dilla University Referral Hospital (58.47%) [7, 15, 22]. Overestimation of the severity of illness may be the main reason for such an empirical use of antimicrobials within 48 hours of admission. Antibiotic were prescribed in conditions with infective etiology use of antibiotic was justified in all cases.

The percentage of encounters in which an injection was prescribed in this study was 44.8%, which was higher than the standard (13.4%–24.1%) derived to serve as ideal [7, 11]. Possible reasons for the high use of injections was our study setting in a referral hospital where patients with serious conditions are treated, and injectable forms produce faster onset of action, and this study was conducted in the emergency setup. The finding was also higher than the study at Ayder Referral Hospital (38.10%) and national baseline study in Ethiopia (23%) and Dilla University Referral Hospital (6.53%) [7, 15, 22]. Injections are very expensive compared to other dosage forms and require trained personnel for administration.

The percentage of drugs prescribed from the essential drug list in this study was 90.2%, which was lower than the standard derived to serve as ideal [7, 11]. It is less than the finding in the same studies conducted in the Hawassa University Hospital (96.60%) in the eastern part of the country (92%) and Adama hospital (94.70%) [16, 23, 24]. It is also less than the studies conducted in Asmera, Eritrea (98.39%), and Saudi Arabia (99.2%) [18, 21].

Regarding facility indicators, the study revealed that DRH had a copy of the EDL. This finding was better than the study conducted in Ethiopia at selected health facilities where only one out of the four hospitals had a copy of the EDL, national drug formulary, and STG during the evaluation period, which makes the overall availability 25% [7].

The mean availability of essential key drugs was 64% in this study, which was comparable (65.7%) with a study conducted in four hospitals of South Ethiopia [25].

### 4.1. Limitations

This study should be interpreted cautiously for different reasons. Firstly, we included only 344 samples, which are below the sample size recommended by WHO to conduct this kind of study. Second, as this was a retrospective study, we might have missed unrecorded data. Finally patient care indicators were not well assessed.

## 5. Conclusion

The findings of this study revealed that drug utilization patterns were not optimal in accordance with the standard values of WHO prescribing indicators. Some of the prescribing indicators and health facility indicators showed deviation from the standard recommended by WHO. Overprescribing of antibiotics and injections, underuse generic drugs, and polypharmacy are the major problems. Therefore, it is very imperative for the concerned stakeholders and healthcare providers to work toward ensuring drug use according to the standard.

## Figures and Tables

**Table 1:** Sociodemographic characteristics of the patients in the emergency department of DRH.

Parameters	Category	Number	Percentage
Age	<14	98	28.49
	14–65	240	69.77
	>65	6	1.74

Gender	Male	211	61.34
	Female	133	38.66

**Table 2:** Commonly prescribed drug classes at the emergency department of DRH.

Drug class	Number	%
CV drug	245	32.54
Antibiotics	208	27.62
NSAID	95	12.62
CNS drugs	73	9.69
GIT drugs	64	8.50
Antidiabetic	58	7.70
Other	10	1.33

**Table 3:** Prescribed drug-related information at the emergency department of DRH.

Prescribed drug-related information	Indicated parameters of prescribed drugs	
	Number	%
Drug name	753	100.00
Strength	717	95.22
Frequency	700	92.96
Dose	685	90.97
Route	682	90.57
Duration	555	73.71

**Table 4:** Drug-prescribing indicators compared WHO drug-prescribing indicators in the emergency department of DRH.

Prescribing indicators	Number	Percent/average	WHO ideal values
Average number of drugs per encounter	753	2.19	(1.6–1.8)
Percentage of encounter with antibiotics	95	27.62	(20.0–26.8%)
Percentage of encounters with injection	154	44.77	(13.4%–24.1%)
Percentage of drugs prescribed by generic	685	90.97	100%
Percentage of drugs from essential drug list	679	90.17	100%

**Table 5:** Number of prescribed drugs per prescription at the emergency department of DRH.

Number of drugs per prescription	Frequency	Percent
One	91	26.45
Two	148	43.02
Three	63	18.32
Four	33	9.59
Five	9	2.62
Total	344	100.0

## Data Availability

The data used to support the findings of this study are available from the corresponding author upon request.

## Abbreviations

CV: Cardiovascular
DM: Diabetes mellitus
DRH: Dilchora referral hospital
EDL: Essential drug list
GIT: Gastrointestinal tract
INRUD: International network for the rational use of drugs
NSAIDs: Nonsteroidal anti-inflammatory drugs
RDU: Rational drug use
RTI: Respiratory tract infection
SNNPR: South nations, nationalities, and people's region
WHO: World health organization.
